# Phyto-nanotechnology in anti-aging medicine

**DOI:** 10.18632/aging.203026

**Published:** 2021-04-27

**Authors:** Alexander Vaiserman, Alexander Koliada, Oleh Lushchak

**Affiliations:** 1 Institute of Gerontology, Kyiv, Ukraine; 2 Molecular Genetic Laboratory Diagen, Kyiv, Ukraine; 3 Vasyl Stefanyk Precarpathian National University, Ivano-Frankivsk, Ukraine

**Keywords:** phytochemicals, aging-related disorders, bioavailability, nanodelivery

Population aging represents a major public health problem worldwide, because current trend in the rise of life expectancy is not accompanied by an increase in healthspan. This is since aging *per se* is a primary risk factor for most pathological conditions related to age. Therefore, developing therapeutic modalities to target processes contributing to aging becomes a priority task for the scientific community.

Phytochemicals offer great hope for the development of new drug classes for treating aging-associated conditions. These compounds may activate pathways involved in aging, such as autophagy, DNA repair, and counteract aging-related systemic oxidative stress and inflammation [1]. Anti-aging potential was reported for several phytobioactive compounds including curcumin, resveratrol, quercetin, epigallocatechin gallate, berberine and several others [2]. The therapeutic potential of orally administered phytochemicals is, however, significantly limited because of their low gastrointestinal absorption, chemical instability, low hydrophilicity, scarce biodistribution and poor penetration/accumulation in the body [3]. These features certainly lead to decreasing the rate and extent of the absorption of oral drugs from solid dosage forms and reducing their bioavailability in the body.

Over the past two decades, innovative nanotechnological applications were developed for delivery of phytochemicals in order to enhance their bioavailability following oral administration. Such nanoformulations, including nanoemulsions, nanoliposomes, nanopolymersomes, nanocrystals, and lipid and polymeric nanoparticles, provide multiple benefits over conventional formulations. These benefits include enhanced solubility and stability in the body, improved absorption in the gastrointestinal tract, protecting from premature enzymatic degradation and metabolism, prolonging the circulation time and minimizing side effects [4]. Available evidence indicates that bioavailability of phytobioactive compounds loaded into nanocarriers can be 5 to 10 times higher than that of their native counterparts [5].

The potential of nanotechnological applications in treating age-related metabolic disorders has been repeatedly confirmed in both *in vitro* and *in vivo* models. Nanodelivery systems loaded with phytobioactive compounds have been consistently confirmed to have a potential to modulate oxidative stress and inflammation known to be important players in aging-associated pathological conditions [6]. Over several recent years, convincing evidence was also reported that phytochemical-loaded nanocarriers can be highly effective in counteracting age-related diseases including cardio-metabolic and neurodegenerative disorders, rheumatoid arthritis and osteoporosis.

Evidence for safety and efficacy of such nanocomposites has been obtained in preclinical animal models (for detailed review, see [3]). In these models, orally administered nano-phyto-formulations showed a more powerful potential to counteract cardio-metabolic disorders in comparison to that of their native forms. Anti-diabetic effects were observed in db/db diabetic mice administered with nanoparticles loaded with berberine. This intervention led to a substantial suppression of body weight gain, improved glucose tolerance and insulin sensitivity. Nanotechnology-based phytotherapy was also shown to be a promising solution in treating rheumatoid arthritis, a chronic autoimmune disease caused due to age-related decline of immune function. In adjuvant-induced arthritis rat models, administration of piperine-loaded solid lipid nanoparticles caused significant decrease of pro-inflammatory cytokine TNFα levels, while treatment with curcumin-loaded solid lipid nanoparticles ameliorated adjuvant-triggered arthritis. Protective effects of curcumin against the bone loss were found to be potentiated by curcumin-loaded nanoparticles in ovariectomized rats. Quercetin-loaded solid lipid nanoparticles were more efficient in restoring bone mineral density in osteopenic animals than native quercetin. Nanobiotechnology-based approaches were also shown to have a great potential in anti-cancer therapy. In various animal models, anti-cancer properties have been shown for nanoparticles loaded with resveratrol, curcumin, EGCG, quercetin and berberine. Importantly, due to such approaches, druggable agents may be delivered directly to tumor sites without damaging nearby healthy tissues.

**Figure 1:**
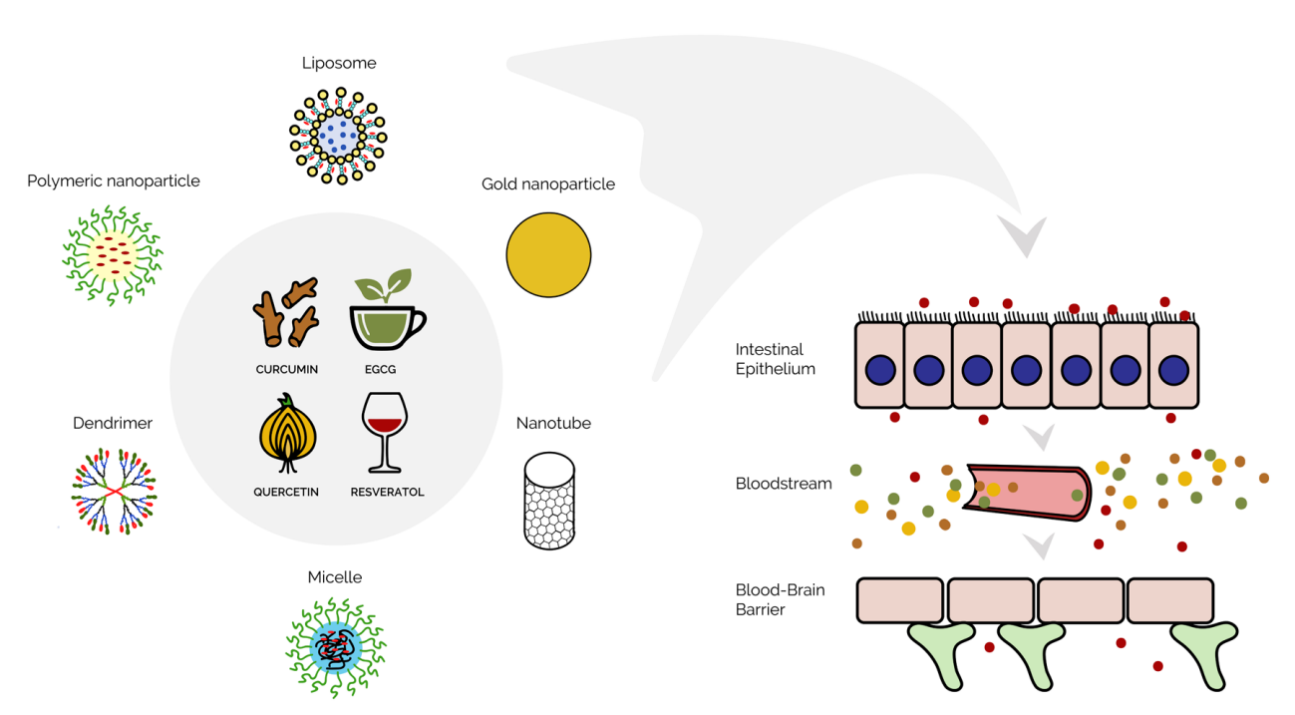
Schematic representation of the nanodelivery of phytochemicals to the brain.

The efficiency of nanodelivery systems was also demonstrated in therapy of age-related neurodegenerative disorders such as Alzheimer’s and Parkinson’s diseases. Developing such applications seems to be particularly important, because the blood-brain barrier is an essential obstacle in delivering pharmaceuticals to the brain. Tunable biophysical features of nanocomposites allowing them to penetrate blood-brain barrier make them highly promising in these therapeutic applications (see Figure 1 for the schematic representation). In particular, enhanced oral bioavailability of curcumin- and resveratrol-loaded solid lipid nanoparticles to the brain has been shown relative to native forms of these compounds. Improved bioavailability to the brain was also observed following administration of some nanocomposites by intranasal or intravenous routes. In a rat model of experimentally induced Alzheimer’s disease, enhanced quercetin delivery to the brain and improved antioxidant effect to brain cells along with improved memory retention were found after treatment with quercetin-loaded solid lipid nanoparticles compared to those of native quercetin. In the same rat model, therapeutic effects of piperine-loaded nanoparticles on Alzheimer’s disease progression, supposedly by reducing oxidative stress and cholinergic degradation, were also observed. Moreover, in rats, EGCG-loaded nanoparticles were shown to attenuate aluminum chloride-induced adverse neurobehavioral impairments via reducing the formation of neurofibrillary tangles and neuritic plaques. Therapeutic potential of resveratrol-loaded solid lipid nanoparticles was also reported in a mouse model of Alzheimer's disease [7]. Nanobiotechnology-based approaches also exhibited therapeutic potential in treating Parkinson’s disease. For instance, in a mouse model of Parkinson’s disease, resveratrol-loaded nanoparticles showed neuroprotective effects against the neurotoxin-caused neurochemical and behavioral impairments.

Summarizing, it can be concluded that using nanodelivery systems holds great promise in phytotherapy of aging-associated disorders. However, several important challenges remain to be addressed. In particular, burst release of therapeutics from nanocarriers may cause cellular toxicity, while slow release can lead to insufficient therapeutic activity. Therefore, development of nanoformulations with optimized release profiles represents an urgent task now [8]. Further research is needed to improve efficiency, safety and cost-effectiveness of nanosized phytodelivery systems prior to widespread clinical application.
